# TGEV activates RIG-I/IFN-β/STAT1 axis to promote NLRC5-mediated SLA-I upregulation

**DOI:** 10.1186/s13567-026-01715-z

**Published:** 2026-03-09

**Authors:** Wenqian Wang, Mengyao Ma, Haojie Bai, Yinhe Sun, Borui Liu, Ao Gao, Qihao Pan, Dongfang Zheng, Muzi Li, Weili Jiang, Shijie Ma, Zhanyong Wei, Honglei Zhang, Lanlan Zheng

**Affiliations:** 1 College of Veterinary Medicine, Henan Agricultural University, Zhengzhou, 450046 China; 2 Food Laboratory of Zhongyuan, Luohe, 462300 China; 3 Ministry of Education Key Laboratory for Animal Pathogens and Biosafety, Zhengzhou, 450046 China; 4 Henan Province Key Laboratory for Animal Food Pathogens Surveillance, Zhengzhou, 450046 China; 5 Longhu Laboratory of Henan Province, Zhengzhou, 450046 China

**Keywords:** Porcine transmissible gastroenteritis virus, SLA-I, NLRC5, viral proliferation

## Abstract

Porcine transmissible gastroenteritis virus (TGEV) is a major pathogen causing acute diarrhea and high mortality in neonatal piglets, yet the interplay between TGEV infection and host immune responses, particularly swine leukocyte antigen class I (SLA-I) mediated antigen presentation and type I interferon (IFN) signaling, remains poorly defined. This study aimed to clarify how TGEV modulates SLA-I expression through NOD-like receptor family CARD domain containing 5 (NLRC5) and the underlying signaling pathways. Since TGEV infection was shown to induce SLA-I upregulation in porcine intestinal tissues, we used swine testicular (ST) cells to investigate the expression dynamics of retinoic acid inducible gene I (RIG-I)/IFN-β/signal transducer and activator of transcription 1 (STAT1) axis. TGEV infection significantly upregulated SLA-I and NLRC5 in ST cells, with high expression coinciding with active viral replication, while NLRC5 inhibited TGEV proliferation. Furthermore, TGEV upregulated SLA-I and NLRC5 expression via RIG-I activation, which triggered downstream IFN-β secretion and subsequent STAT1 phosphorylation, thereby facilitating the induction of SLA-I and NLRC5. Moreover, the TGEV open reading frame 7 (ORF7) protein was identified as a critical viral effector contributing to SLA-I and NLRC5 upregulation. Collectively, TGEV infection activates the RIG-I/IFN-β/STAT1 axis to promote NLRC5 mediated SLA-I upregulation, which inhibits TGEV proliferation, elucidating a novel immune regulatory mechanism that balances viral replication and host antiviral defense and providing insights for targeted management strategies against porcine enteric coronaviruses.

**Supplementary Information:**

The online version contains supplementary material available at 10.1186/s13567-026-01715-z.

## Introduction

Transmissible gastroenteritis virus (TGEV) is an enveloped, single-stranded RNA virus belonging to the *Coronaviridae* family. In newborn piglets, infection is characterized by severe acute diarrhea and vomiting, resulting in a 100% mortality rate and causing substantial economic losses to the swine industry. TGEV is highly contagious that widely distributed all over the world [1], and the positive rate can reach up to 38% in some areas of China with the average infection rate of 10% from 1983 to 2022 [2]. Although the overall prevalence has declined in recent years, many regions continue to report TGEV antibody-positive cases [3]. A recent study demonstrated that the receptor-binding domains of TGEV can interact with aminopeptidase N from eight nonporcine animal species, suggesting a potential risk of cross-species transmission [4]. These findings highlight the importance of sustained vigilance and strengthened preventive measures against TGEV.

The genome of TGEV includes nine open reading frames (ORFs), arranged in the order 5′-ORF1a-ORF1b-Spike glycoprotein (S)-ORF3a-ORF3b-Envelope protein (E)-Membrane glycoprotein (M)-nucleocapsid protein (N)-ORF7-3′ [5]. Accessory proteins of viruses, including those of coronaviruses, are known to play crucial roles in the viral life cycle, significantly influencing replication and virulence [6–8]. The TGEV ORF7 protein, encoding a 78-amino-acid hydrophobic protein (HP) with a molecular weight of approximately 9.1 kDa, has been implicated in viral replication and assembly [9, 10]. Beyond its role in TGEV, studies on homologous proteins in related coronaviruses suggest that ORF7 can suppress the production of interferon (IFN) in vitro and modulate the chemotactic responses of monocytes and neutrophils, as observed in severe acute respiratory syndrome coronavirus-2 (SARS-CoV-2) [11, 12].

The major histocompatibility complex (MHC) encodes and produces a class of proteins called antigen-presenting molecules, which are pivotal in mediating immune responses [13]. In swine, MHC molecules are known as swine leukocyte antigens (SLA) [14]. MHC molecules are categorized into MHC-I and MHC-II based on their structural and functional differences. MHC-I is expressed on all nucleated cells, albeit at varying levels across tissues and cell types, and mediates antiviral defense by presenting endogenous antigens to CD8^+^ T lymphocytes, thereby facilitating the immune recognition and clearance of virus-infected or malignant cells [13, 15]. In contrast, MHC-II is predominantly expressed on the surface of professional antigen-presenting cells (APCs), where it presents processed exogenous antigens to CD4^+^ T lymphocytes [16]. In response to pathogenic threats or cellular stressors, the immune system generates IFN, which subsequently upregulate MHC-I expression on APCs to enhance pathogen clearance [17]. Conversely, many viruses, including porcine epidemic diarrhea virus (PEDV), SARS-CoV-2, and Middle East respiratory syndrome coronavirus (MERS-CoV), evade host immune surveillance by downregulating the expression or surface presentation of MHC-I, thereby escaping recognition by CD8^+^ T cell [15, 18, 19]. In addition, accessory proteins such as ORF7a and ORF3a of SARS-CoV-2 have been shown to modulate MHC-I surface expression to facilitate immune evasion [15]. Nevertheless, the role of MHC-I in mediating host immune responses against TGEV infection remains to be fully elucidated.

As a cytosolic pattern recognition receptor (PRR), retinoic acid-inducible gene I (RIG-I) detects viral RNA within the cytoplasm, initiating a signaling cascade that culminates in the secretion of type I interferon (IFN-I) [20]. In RIG-I-mediated antiviral responses, MHC-I molecules on the surface of infected cells present viral peptides to activate CD8^+^ T cells [21]. Simultaneously, RIG-I signaling enhances antigen presentation by upregulating MHC-I expression in dendritic cells (DCs), thereby potentiating their ability to prime CD8^+^ T cell responses [22]. This process facilitates the display of abundant viral antigens, further amplifying cytotoxic T lymphocyte activation and strengthening antiviral immunity. The engagement of peptide-MHC complexes with their cognate T cell receptors triggers intracellular signaling pathways that activate key transcriptional regulators [23], thereby modulating cellular gene expression. During antigen presentation, for instance, signal transducer and activator of transcription 1 (STAT1) translocates into the nucleus and bind to specific promoter regions to regulate immune-related gene transcription [24].

As a recently identified and physiologically crucial member of the NOD-like receptor (NLR) family, NLR family CARD domain containing 5 (NLRC5) contains structural domains homologous to apoptosis-associated signal transduction proteins [25]. The promoter region of the *NLRC5* gene harbors several conserved regulatory elements, including binding sites for STAT1, nuclear factor kappa-B (NF-κB), and IFN response elements, which are essential for governing its transcription [26–28]. The presence of these IFN-responsive motifs suggests that IFN signaling may directly initiate NLRC5 transcription. Notably, NLRC5 functions as a key transcriptional regulator of MHC-I gene expression. Following cellular detection of pathogens or inflammatory stimuli, *NLRC5* is upregulated in various cell types and tissues, where it participates in initiating immune responses [28]. Mechanistically, NLRC5 activates MHC-I transcription by binding specifically to cis-regulatory sequences within MHC-I gene promoters [29]. Both the N-terminal effector domain and the C-terminal leucine-rich repeat (LRR) domain of NLRC5 are essential for this transactivation function, and its overexpression is sufficient to elevate MHC-I expression levels [30]. Viruses have evolved strategies to counteract this pathway. The nonstructural protein 1 (Nsp1) protein of PEDV inhibits NLRC5 translation to subvert MHC-I-mediated immunity [18]. This is particularly relevant as Nsp1, encoded within the ORF1a/b region of coronavirus, is an established key virulence factor [31]. Given these interactions, the precise regulatory interplay between TGEV infection and the expression dynamics of MHC-I and its transcriptional regulator NLRC5 warrants further investigation.

In this study, we observed that TGEV infection significantly upregulates SLA-I expression through a mechanism mediated by the concurrent induction of NLRC5. Further investigation revealed that the RIG-I/IFN-β/STAT1 signaling axis plays a crucial role in mediating the host response to TGEV infection. This upregulation of SLA-I expression was confirmed in both porcine intestinal tissues and ST cells upon TGEV challenge. Utilizing the ST cell model, we elucidated the mechanism underlying TGEV–host interactions and their modulation of innate immune pathways. Moreover, the TGEV-encoded ORF7 protein was identified as a critical viral factor responsible for enhancing the expression of both SLA-I and NLRC5 in infected cells. These findings uncover pivotal mechanisms by which TGEV may modulate host immunity and provide a foundation for developing targeted f antiviral strategies.

## Materials and methods

### Virus and cell cultures

The viral strain used in this study was TGEV HN-2012 (GenBank accession number: OP434397.1), with a titer of 10^8.0^ TCID_50_/0.1 mL. This isolate was obtained and maintained in our laboratory. To prepare ultraviolet-inactivated TGEV (UV-TGEV), the viral suspension was exposed to UV irradiation for 12 h. Complete loss of viral infectivity was confirmed by the cytopathic effect (CPE) and through immunofluorescence assay (IFA). ST cells were cultured in Dulbecco’s Modified Eagle Medium (DMEM, Gibco, USA) supplemented with 10% heat-inactivated fetal bovine serum (FBS, Gibco, USA) and were maintained at 37 ℃ with 5% CO_2_.

### RNA extraction and characterization

RNA was extracted from colon, duodenum, jejunum, and ileum tissues obtained from TGEV-positive piglet samples maintained in our laboratory. For in vitro infection, ST cells (1 × 10^6^ cells/well) were infected with TGEV at a multiplicity of infection (MOI) of 1, followed by RNA isolation. Total RNA was extracted using TRIzol Reagent (Vazyme, China) according to the manufacturer’s instructions, and cDNA was then synthesized from the extracted RNA using the HiScript II 1 st Strand cDNA Synthesis Kit (Vazyme, China). The expression levels of target genes were quantified by reverse transcription quantitative PCR (RT-qPCR). The mRNA level of β-actin, amplified from the same cDNA, was used as an endogenous control to normalize for total RNA input. The sequences of all primers used are listed in Table 1.

**Table 1:** **List of primers used in the study**

Primer name	Accession numbers in Genbank	Sequence (5′–3′)
TGEV	OP434397.1	F: TGAAGGGCCAACGTAAAGAG
		R: CAACCCAGACAACTCCATCTAA
NLRC5	MT773565	F: TCCAAACAAGTGCGATGA
		R: TGAGCCAGTTCCCAGATT
SLA-1	KU754558.1	F: GTGGCTGGAGTTGTGATC
		R: ACCCTTGGTAAGGGACAC
SLA-2	KU754573.1	F: AGGGAGAGAGGAGCTACC
		F: ATGTGTCTTTGGAGGCTC
SLA-3	KU754583.1	F: CACAGACTTTCCGAGTGA
		R: TAGGCGTCCTGACTGTAC
β2M	AF452448.1	F: CCTGTCTTTCAGCAAGGA
		R: CGGTTAGTGGTCTCGATC
TAP1	DQ227991.1	F: ACGGGGACTGTGTCTCTT
		R: GAGATTCCTGCACCTGTG
PSMβ9	DQ295031.1	F: GCCGTCATTTTCTGCCTCATC
		R: TAGGCTCGCAGGGATGATTTC
STAT1	HQ450761.1	F: AGGCATCGCCGACAGGA
		R: TCTCACGGTTGGCTTTGG
RIG-I	MF358966.1	F: GGCTGAAGCCACAGAATA
		R: TCAGTGGTCCGTAATTCC
ORF1a	UXN48786.1	F: ATGAGTTCCAAACAATTCAAGATCC
		R: TTAGAGTTCTTTTTGACTGGTGGTG
ORF3b	UXN48788.1	F: ATGATTGGTGGACTTTTTCTTAATACTC
		R: CTAGGAAACGTCATAGGTACGGTCT
ORF7	UXN48792.1	F: CGTCCTCCTCGATGCTGTATT
		R: GAAAGATTAATCGAAGCACCACT
β-actin	ON164673.1	F: GGTATCAGCAGCAGTCTTA
		R: TTCACAGAGGCGAGTAACTT

### Western blot analysis

The ST cell samples were collected and lysed in RIPA buffer (Solarbio, China) containing 1% protease inhibitor cocktail (MCE, China) for 20 min on ice, followed by denaturation at 100 °C for 10 min. Electrophoretic fractionation of proteins was carried out on 12% polyacrylamide gels and subsequently transferred onto nitrocellulose membranes. The membranes were blocked in 5% skim milk prepared in Tris-buffered saline with Tween-20 (TBST) at 25 ℃ for 2 h, then incubated overnight at 4 ℃with the following primary antibodies: mouse anti-SLA-I monoclonal antibody (Novus Biologicals, USA, 1:400), mouse anti-β-actin monoclonal antibody (proteintech, China, 1:1000), monoclonal antibody against TGEV-N protein (provided by our laboratory), STAT1 monoclonal antibody (Proteintech, China, 1:5000), and RIG-Ⅰ/DDX58 polyclonal antibody (Proteintech, China, 1:1000). For subcellular localization analysis, ST cells were cultured to approximately 80% confluence in 12-well plates and infected with TGEV at an MOI of 1. Nuclear and cytoplasmic fractions were isolated using a Nuclear and Cytoplasmic Protein Extraction Kit (Beyotime, China) to examine NLRC5 localization. In separate experiments, uninfected ST cells were treated with recombinant human IFN-β protein (Univ, China) and Anifrolumab (MCE, China) at concentrations of 10, 100, and 1000 ng/mL for 24 h. These samples were lysed in RIPA supplemented with 1% protease inhibitors on ice for 20 min, denatured at 100 ℃ for 10 min, and subjected to electrophoresis on 12% polyacrylamide gels. After transfer, membranes were incubated overnight at 4 ℃ with primary antibodies: mouse anti-SLA-I monoclonal antibody (Novus Biologicals, USA, 1:400), mouse anti-β-actin monoclonal antibody (proteintech, China, 1:1000), monoclonal antibody against TGEV-N protein (provided by our laboratory), and the NLRC5 polyclonal antibody provided by our laboratory. Following primary antibody incubation, membranes were probed with HRP‑conjugated goat anti-mouse or goat anti-rabbit secondary antibodies (Proteintech, China, 1:4000). Protein bands were visualized using the ECL Prime Western Blot Detection Reagent (Promega, USA) and imaged with Amersham Imager 680 system (GE, USA).

### Construction of recombinant plasmids

Recombinant plasmids encoding NLRC5, ORF1a, ORF3b, ORF7, STAT1, and RIG-I were constructed through PCR amplification of the respective target genes using the primer pairs listed in Table 1. The resulting amplicons were purified and subsequently cloned into the pCAGGS-HA vector for NLRC5, ORF1a, ORF3b, ORF7, and RIG-I, or the pCAGGS-FLAG vector for STAT1 using the ClonExpress II One Step Cloning Kit (Vazyme, China). The validated plasmids were amplified in *Escherichia coli* DH5α competent cells (Tsingke, China), and then transfected into ST cells using Cellfectin® II Reagent (Gibco, USA) or Lipofectamine 2000 reagent (Invitrogen, USA) according to the manufacturer’s instructions. After 36 h post-transfection, cells were harvested and lysed. Recombinant protein expression was analyzed by western blotting with a mouse anti-HA monoclonal antibody (Proteintech, China; 1:1000) as the primary antibody, followed by an HRP-conjugated goat anti-mouse IgG secondary antibody (Proteintech, China; 1:4000). Protein signals were detected using the Amersham Imager 680 system.

### Detection of NLRC5 promoter activity

The Dual-Luciferase Reporter Assay System (Promega, Madison, WI, USA) was used to evaluate the promoter activity of NLRC5. ST cells were co-transfected with the NLRC5 promoter-driven firefly luciferase reporter plasmid and the pRL-TK Renilla luciferase control plasmid using Lipofectamine 2000 reagent for 12 h. Subsequently, cells were either infected with TGEV at an MOI of 1 or transfected with the pCAGGS-HA-RIG-I or pCAGGS-FLAG-STAT1 expression plasmids, and harvested 24 h later. After washing with phosphate buffered saline (PBS), cells were lysed with 60 µL/well of RIPA buffer containing 1% protease inhibitors for 20 min at 25 ℃. Luciferase activity was measured by sequentially adding 100 µL of LAR II reagent to quantify firefly luciferase signal, followed by 100 µL of Stop and Glo reagent to quantify Renilla luciferase activity. The firefly-to-Renilla luciferase activity ratio was calculated for normalization and data analysis.

### Synthetic siRNAs and transfection

The siRNA sequences targeting NLRC5, STAT1, and RIG-I were designed and synthesized by GenePharma (China), with their sequences listed in Table 2. ST cells were seeded in 6-well plates and grown to approximately 70% confluence before transfection. siRNA transfection was performed using Lipofectamine 2000 reagent at concentrations of 10, 20, 30, and 40 pmol/µL. At 24 h post-transfection, cells were infected with TGEV at an MOI of 1, and total RNA and protein were harvested at 24 h postinfection (hpi) for subsequent RT-qPCR and western blot analyses.

**Table 2:** **Sequences of sense strands for SiRNA**

SiRNA name	Sequence (5′–3′)
NLRC5-Sus-1	F: GGAGGAGAAUGCUAAUAAATT
	R: UUUAUUAGCAUUCUCCUCCTT
NLRC5-Sus-2	F: GAGGCUAGGAAGACACUAATT
	R: UUAGUGUCUUCCUAGCCUCTT
NLRC5-Sus-3	F: CCAGGAGUUUGCCAACAAUTT
	R: AUUGUUGGCAAACUCCUGGTT
STAT1-Sus-1	F: CUGACAUUAUUCGCAAUUATT
	R: UAAUUGCGAAUAAUGUCAGTT
STAT1-Sus-2	F: GGUGUAUUGUGGGCUUUAUTT
	R: AUAAAGCCCACAAUACACCTT
STAT1-Sus-3	F: GCGUAACCUUCAGGAUAAUTT
	R: AUUAUCCUGAAGGUUACGCTT
RIG-I-Sus-1	F: GACCCUAGAAGAUCUACAATT
	R: UUGUAGAUCUUCUAGGGUCTT
RIG-I-Sus-2	F: CAGCCCUGGAAGAAAUCAUTT
	R: AUGAUUUCUUCCAGGGCUGTT
RIG-I-Sus-3	F: GCAGGAGGAAGCCAACAAUTT
	R: UUUGUUGUCUAACUCCUGGTT

### Immunofluorescence assay

Upon reaching 70% confluence, ST cells were transfected with either 2 µg of the NLRC5 recombinant plasmid, pCAGGS-HA vector, or 20 pmol/µL of siNLRC5#2. At 24 h post‑transfection, the cells were infected with TGEV at an MOI of 1. Subsequently, cells were fixed overnight at 4 ℃ with anhydrous ethanol and permeabilized with 0.05% Triton X-100 (Solarbio, China) at 25℃ for 5 min. After rinsing with PBS, samples were blocked with 5% BSA (Gibco, USA) in PBS at 37 ℃ for 2 h. For immunofluorescence staining, cells were incubated overnight at 4 ℃ with a mouse anti-TGEV-N primary antibody (1:400), followed by a 1 h incubation at 37 ℃ with fluorescein isothiocyanate (FITC)-conjugated goat anti-mouse IgG (Sigma-Aldrich, USA, 1:200). Nuclei were counterstained with 4′,6-diamidino-2-phenylindole (DAPI; Sangon Biotech, China) at 37 ℃ for 10 min, and fluorescence images were obtained using an Olympus instrument (Japan).

### TGEV replication in NLRC5-overexpressing cells

ST cells were plated in 12-well plates and grown to 70% confluence before transfection with either the pCAGGS-HA-NLRC5 plasmid or 20 pmol/µL siNLRC5#2 for 24 h. Cells were then inoculated with TGEV at an MOI of 1 for 1 h at 37 ℃, followed by the addition of 2 mL maintenance medium each well. Samples were harvested at 6, 12, 18, 24, and 36 hpi and subjected to three cycles of freeze−thaw at −80 ℃. Viral titers were determined by the 50% tissue culture infective dose (TCID_50_) assay, and viral RNA levels were quantified by RT-qPCR as previously described [32, 33].

### Confocal immunofluorescence assay

ST cells were transfected with the pCAGGS-HA-NLRC5 plasmid using Lipofectamine 2000 reagent. After 24 h post-transfection, cells were infected with TGEV (MOI = 1) and harvested at the indicated time points. Cells were fixed overnight at 4 ℃ with anhydrous ethanol, permeabilized with 0.05% Triton X-100 for 5 min at 25 ℃, washed three times with PBS, and blocked with 5% BSA in PBS at 37 ℃ for 2 h. Samples were then incubated overnight at 4 ℃ with a mouse anti-HA monoclonal antibody (1:1000), followed by a 1 h incubation at 37 ℃ with FITC-conjugated goat anti-mouse IgG (1:200). Nuclei were counterstained with DAPI at 37 ℃ for 10 min, and images were acquired using a confocal laser scanning microscope (Olympus, Japan).

### Statistical analysis

Statistical analyses were performed using GraphPad Prism version 9.5. Differences among multiple groups were analyzed by one-way analysis of variance (ANOVA), with significance levels denoted as *p* < 0.05 (*),* p* < 0.01 (**), and* p* < 0.001 (***).

## Results

### TGEV infection upregulates the expression of SLA-I

**Figure 1:**
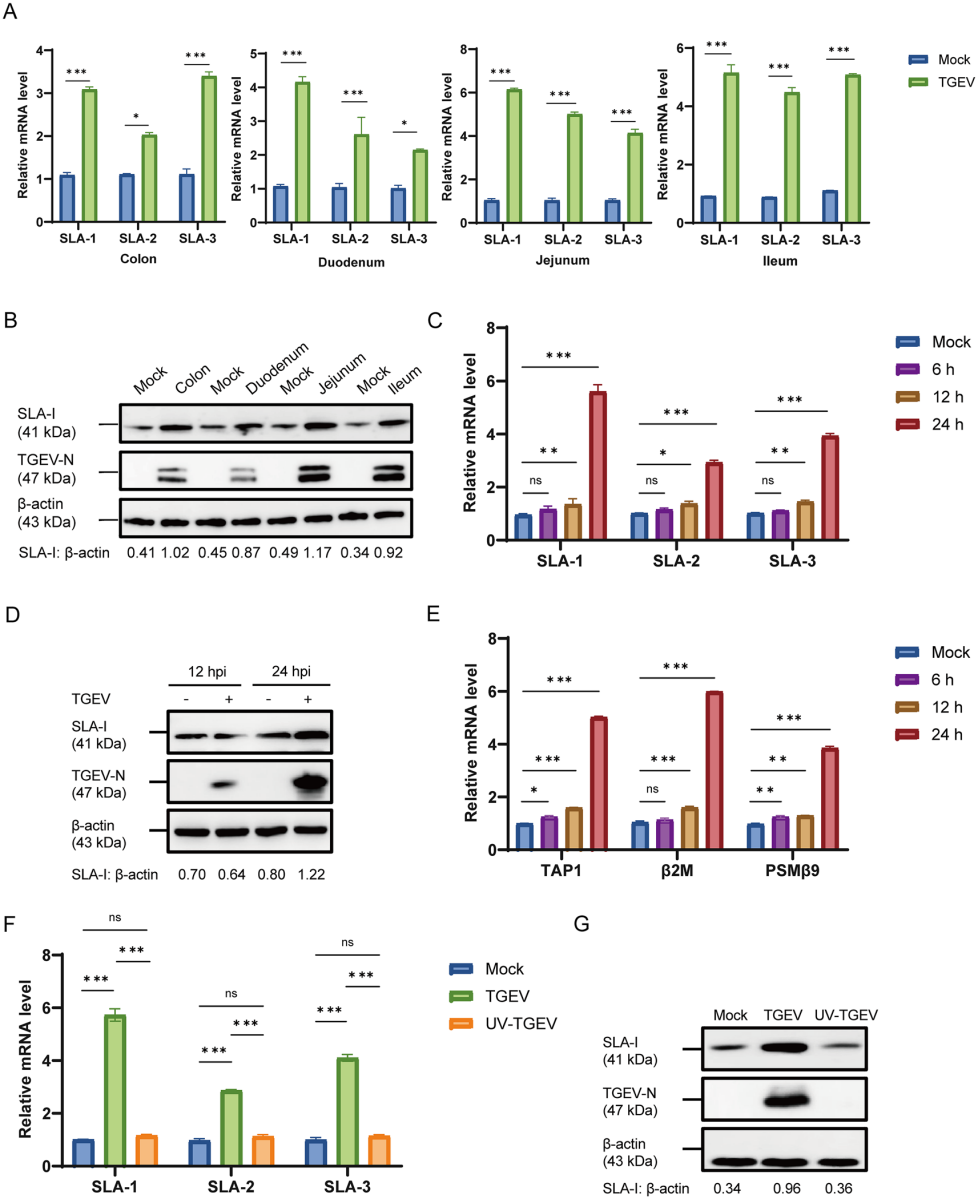
**TGEV infection upregulates the expression of SLA-I (***n*** = 3)**. **A** The relative mRNA levels of SLA-1, SLA-2, and SLA-3 in colon, duodenum, jejunum, and ileum of pigs after TGEV infection were assessed by RT-qPCR. **B** The protein expression of SLA-I in colon, duodenum, jejunum, and ileum of pigs infected with TGEV was detected by western blot. **C** ST cells were infected with TGEV (MOI = 1), and harvested at 6, 12, and 24 hpi. The relative mRNA levels of SLA-1, SLA-2, SLA-3 were quantified by RT-qPCR. **D** The protein expression of SLA-I in ST cells was detected by western blot at 12 and 24 hpi. **E** ST cells were infected with TGEV (MOI = 1), and the relative mRNA levels of TAP1, β2M, and PSMB9 were quantified by RT-qPCR at 6, 12, and 24 hpi. **F**, **G** ST cells were treated simultaneously with either TGEV or UV-TGEV (MOI = 1) and harvested at 24 hpi. The mRNA levels of SLA-1, SLA-2, SLA-3 (**F**), and protein level of SLA-I (**G**) in ST cells were assessed by RT-qPCR and western blot, respectively. *p < 0.05, **p < 0.01, ***p < 0.001.

SLA-I molecules function as primary mediators for presenting endogenous antigens to CD8^+^ T lymphocytes, thereby initiating specific cellular immune responses. To determine changes in SLA-I expression following TGEV infection, we first examined intestinal tissues from TGEV-positive piglet samples maintained in our laboratory. Total RNA extracted from the colon, duodenum, jejunum, and ileum of piglets was subjected to RT-qPCR detection. The results demonstrated that the mRNA levels of SLA-1, SLA-2, and SLA-3 were significantly upregulated in the intestinal tissues of TGEV-infected pigs compared with controls (Figure 1A), confirmed an increase in SLA-I transcription. We further evaluated SLA-I expression at the protein level by western blot. Consistent with the mRNA data, SLA-I protein abundance was markedly elevated in colon, duodenum, jejunum, and ileum tissues from TGEV-infected piglets (Figure 1B). To determine whether TGEV infection also induces SLA-I expression in vitro, ST cells were infected with TGEV (MOI = 1) and samples were harvested at various time points for RT-qPCR analysis. SLA-I transcription levels remained unchanged at 6 hpi but were significantly increased at 12 hpi, with further elevation at 24 hpi (*p* < 0.001) compared with the mock‑infected group (Figure 1C). Correspondingly, western blot analysis of cellular lysates collected at 12 and 24 hpi indicated a significant increase in SLA-I protein levels at 24 hpi, correlating positively with TGEV infection (Figure 1D). These results indicated that TGEV infection triggers a time-dependent upregulation of SLA-I expression. We also examined the expression of key MHC-I-related genes of transporter 1 (TAP1), *β*_2_-microglobulin (*β*2M), and proteasome 20S subunit beta 9 (PSMB9) by RT-qPCR. The findings revealed that their mRNA levels peaked at 24 hpi (*p* < 0.001) (Figure 1E), supporting the coordinated induction of the MHC-I antigen processing pathway. To assess whether the upregulation of SLA-I depends on active viral replication, ST cells were treated with either live TGEV or UV-TGEV for 24 h. Unlike live virus, UV-TGEV failed to enhance SLA-I expression, confirming that the induction of SLA-I is viral replication dependent (Figures 1F and G). Taken together, these findings demonstrate that TGEV infection upregulates SLA-I expression both in animal models and laboratory cell cultures.

### TGEV infection increases SLA-I expression by upregulating NLRC5

**Figure 2:**
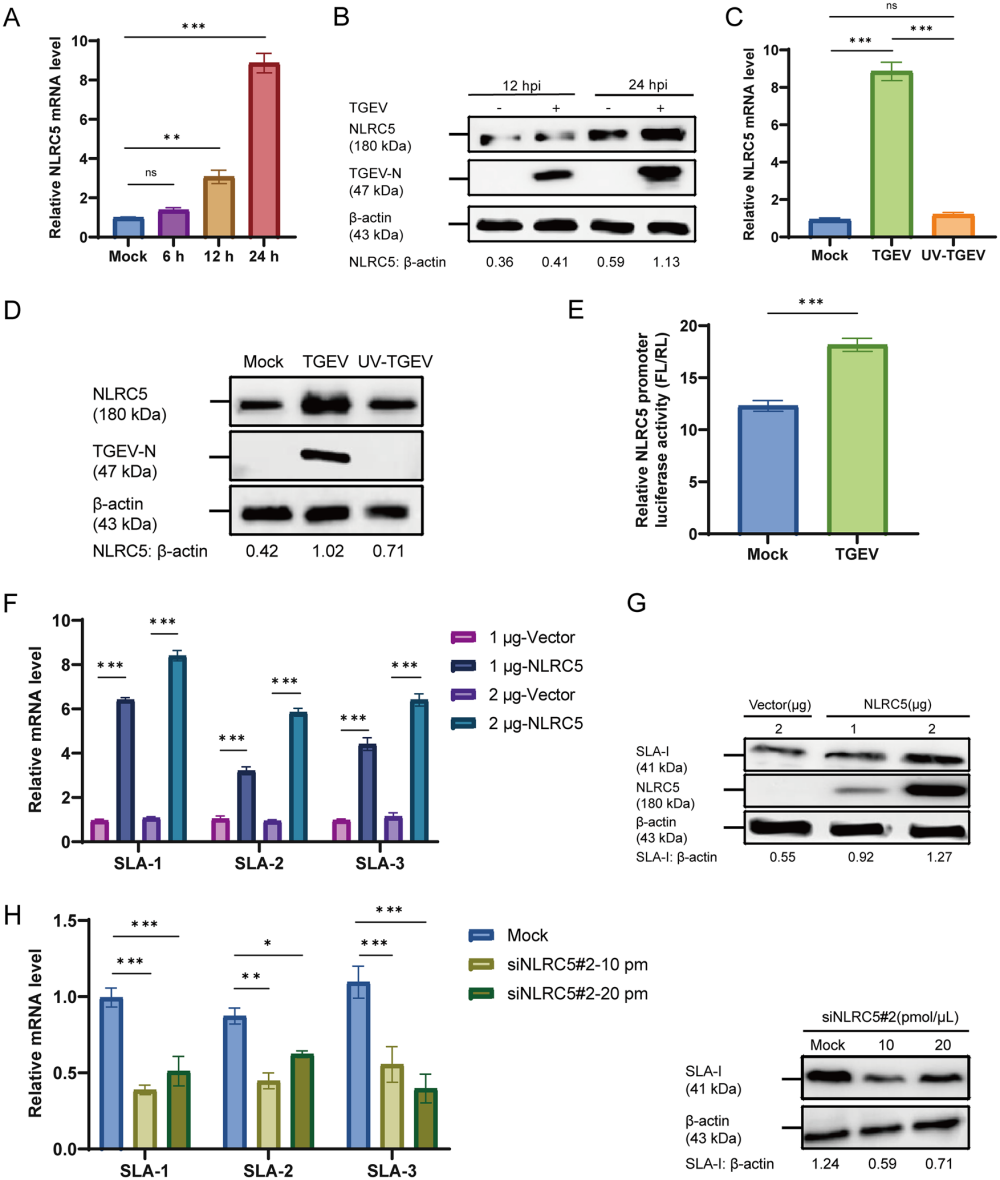
**TGEV infection increases SLA-I expression by upregulating NLRC5 (***n*** = 3)**. **A**, **B** ST cells were infected with TGEV (MOI = 1), and harvested at 6, 12, and 24 hpi. The mRNA (**A**) and protein (**B**) levels of NLRC5 in TGEV-infected cells were assessed by RT-qPCR and western blot, respectively. **C**, **D** ST cells were simultaneously treated with either TGEV or UV-TGEV (MOI = 1) and harvested at 24 hpi. NLRC5 at the mRNA (**C**) and protein (**D**) levels were assessed by RT-qPCR and western blot. **E** ST cells were co-transfected with the NLRC5 promoter-driven dual-luciferase reporter plasmid (500 ng) and the pRL-TK plasmid (50 ng). After 24 h, cells were infected with TGEV (MOI = 1) and luciferase activity was then measured 24 h later using a dual-luciferase assay (FL: firefly luciferase, RL: Renilla luciferase). **F**, **G** ST cells were transfected with pCAGGS-HA (1 or 2 μg) or pCAGGS-HA-NLRC5 (1 or 2 μg). After 24 h, the cells were harvested for analysis. The relative mRNA levels of SLA-1, SLA-2, and SLA-3 (**F**) and the protein level of SLA-I (**G**) were detected by RT-qPCR and western blot at 24 hpi, respectively. **H**, **I** ST cells were transfected with siNLRC5#2 at 10 and 20 pmol/μL for 24 h, followed by TGEV infection (MOI = 1) for an additional 24 h. The relative mRNA levels of SLA-1, SLA-2, and SLA-3 (**H**) and the protein level of SLA-I (**I**) were detected by RT-qPCR and western blot at 24 hpi, respectively. *p < 0.05, **p < 0.01, ***p < 0.001.

NLRC5 functions as a trans-activator of MHC-I. To confirm whether TGEV modulates SLA-I expression through NLRC5, we quantified NLRC5 mRNA levels at 6, 12, and 24 hpi by RT-qPCR and protein levels at 12 and 24 hpi by western blot. Both mRNA and protein levels of NLRC5 increased in a time-dependent manner after infection, suggesting that TGEV infection stimulated NLRC5 induction in ST cells (Figures 2A and B). To investigate whether viral replication is required for NLRC5 induction, ST cells were treated with either live TGEV or UV-TGEV. No significant induction of NLRC5 was observed with UV-TGEV by either RT-qPCR or western blot (Figures 2C and D), confirming that NLRC5 induction depends on TGEV replication. In addition, a luciferase reporter driven by the NLRC5 promoter showed markedly enhanced activity upon TGEV infection (*p* < 0.001), indicating that TGEV infection enhances NLRC5 transcription (Figure 2E). Moreover, we confirmed the expression of the recombinant plasmid pCAGGS-HA-NLRC5 in ST cells (Additional file 1A). Transfection of pCAGGS-HA-NLRC5, but not the empty pCAGGS-HA vector, significantly increased the mRNA levels of SLA-1, SLA-2, and SLA-3 compared with the control (*p* < 0.001; Figure 2F). Furthermore, transfection with increasing amounts (1 or 2 μg) of pCAGGS-HA-NLRC5 resulted in a dose-dependent upregulation of SLA-I protein (Figure 2G). To investigate whether TGEV upregulates SLA-I via NLRC5, we screened siRNA targeting NLRC5 and identified siNLRC5#2 with 10 and 20 pmol/μL as effectively reducing NLRC5 expression relative to negative control (*p* < 0.01) (Additional file 1B and C). ST cells were pretreated with siNLRC5#2 (10 and 20 pmol/μL) for 24 h, followed by TGEV infection. After another 24 h, both mRNA levels of SLA-1, SLA-2, and SLA-3 and SLA-I protein expression were assessed. Compared with controls, both transcriptional and protein levels of SLA-I were downregulated, which demonstrated that the decrease of NLRC5 expression led to a reduction of SLA-I (Figures 2H and I). These data demonstrated that NLRC5 serves as a critical mediator of TGEV-induced upregulation of SLA-I.

### NLRC5 inhibits TGEV proliferation

**Figure 3:**
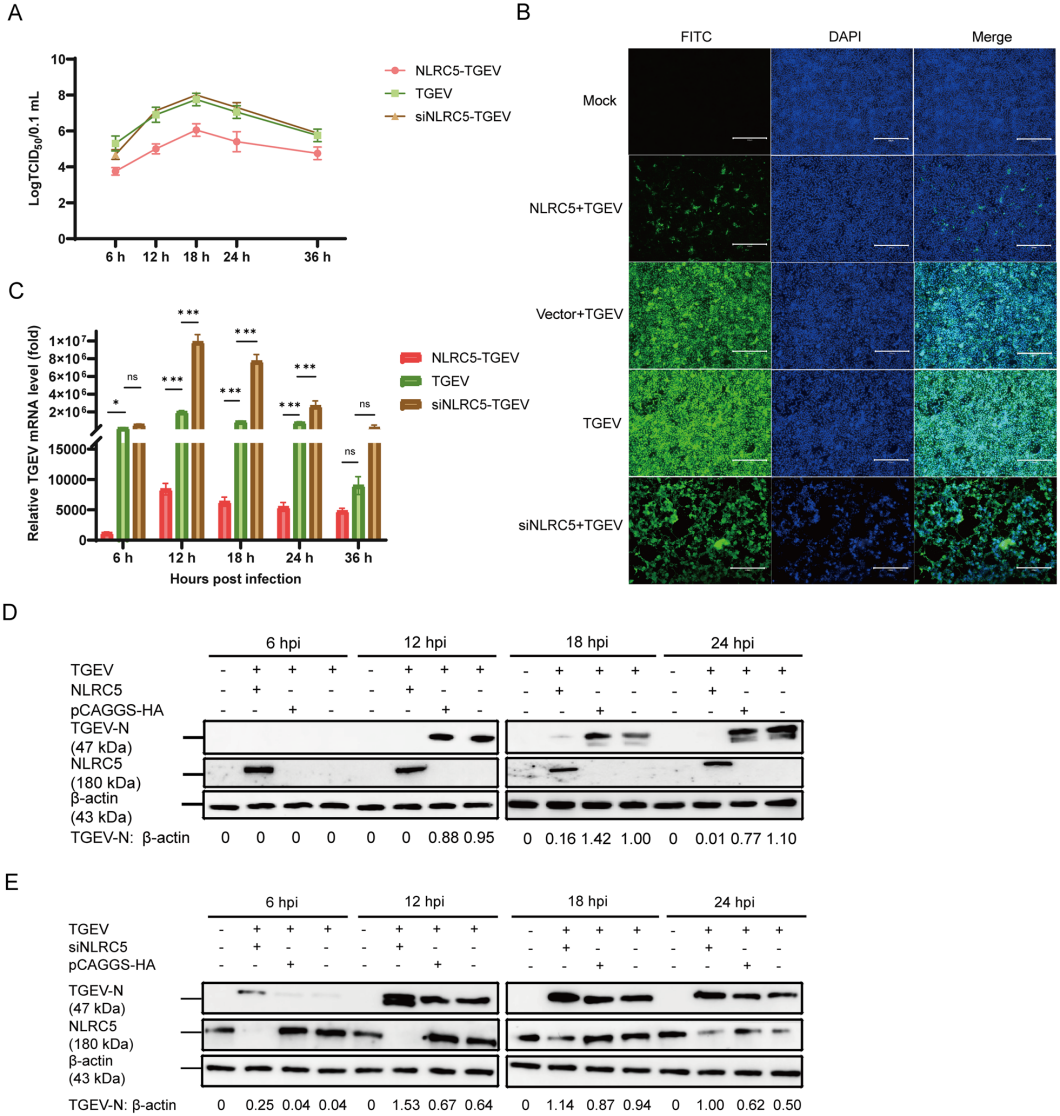
**NLRC5 inhibits the proliferation of TGEV**. **A** ST cells were transfected with either pCAGGS-HA-NLRC5 or siNLRC5#2 (20 pmol/μL) for 24 h, then they were subjected to TGEV infection (MOI = 1). Cells were harvested at 6, 12, 18, 24, and 36 hpi for TCID_50_ determination. **B** ST cells were transfected with the pCAGGS-HA-NLRC5, pCAGGS-HA vector, or siNLRC5#2 (20 pmol/μL) for 24 h, respectively, and then infected with TGEV (MOI = 1). At 24 hpi, cells were harvested and processed for immunofluorescence assays. Scale bar: 300 µm. **C** ST cells were transfected with pCAGGS-HA-NLRC5 or siNLRC5#2 (20 pmol/μL) for 24 h, respectively, then they were subjected to TGEV infection (MOI = 1), and the cell samples were collected to assess TGEV proliferation by RT-qPCR at 6, 12, 18, 24, and 36 hpi (*n* = 3). **D**, **E** ST cells were transfected with pCAGGS-HA-NLRC5, pCAGGS-HA vector, or siNLRC5#2 (20 pmol/μL). After 24 h, cells were infected with TGEV (MOI = 1) and harvested at 6, 12, 18, and 24 hpi for western blot analysis of TGEV proliferation. *p < 0.05, **p < 0.01, ***p < 0.001.

TGEV infection resulted in considerable elevation of both SLA-I and NLRC5 expression in ST cells. ST cells were first transfected with pCAGGS-HA-NLRC5, pCAGGS-HA vector, or with siNLRC5#2 at 20 pmol/µL for 24 h, respectively. After this transfection, the cells were infected with TGEV to examine the role of NLRC5 in TGEV replication, and harvested at 6, 12, 18, 24, and 36 hpi for TCID_50_ determination. Notably, overexpression of NLRC5 significantly reduced viral titers compared with the TGEV group, whereas viral titers in the siNLRC5-TGEV group were comparable to those in the TGEV group (Figure 3A). Consistently, IFA showed markedly diminished green fluorescence in ST cells transfected with NLRC5. In contrast, in ST cells transfected with siNLRC5, CPE were more severe compared with cells infected with TGEV alone at 24 hpi, and accompanied by extensive cell detachment. These results indicate that the suppressive role of NLRC5 in TGEV N protein expression (Figure 3B). Complementary assays by RT-qPCR and western blot confirmed a suppressive impact of NLRC5 on TGEV replication. Specifically, NLRC5 overexpression substantially reduced TGEV mRNA at 12, 18, and 24 hpi (*p* < 0.001) (Figure 3C), and concomitantly decreased TGEV-N protein expression relative to control group (Figure 3D). However, silencing NLRC5 resulted in the opposite effect compared with NLRC5 overexpression that silencing NLRC5 significantly promotes TGEV proliferation (Figures 3C and E). These results demonstrated that NLRC5 suppressing proliferation of TGEV both at transcriptional and translational levels.

### TGEV promotes the nuclear transportation of NLRC5

**Figure 4:**
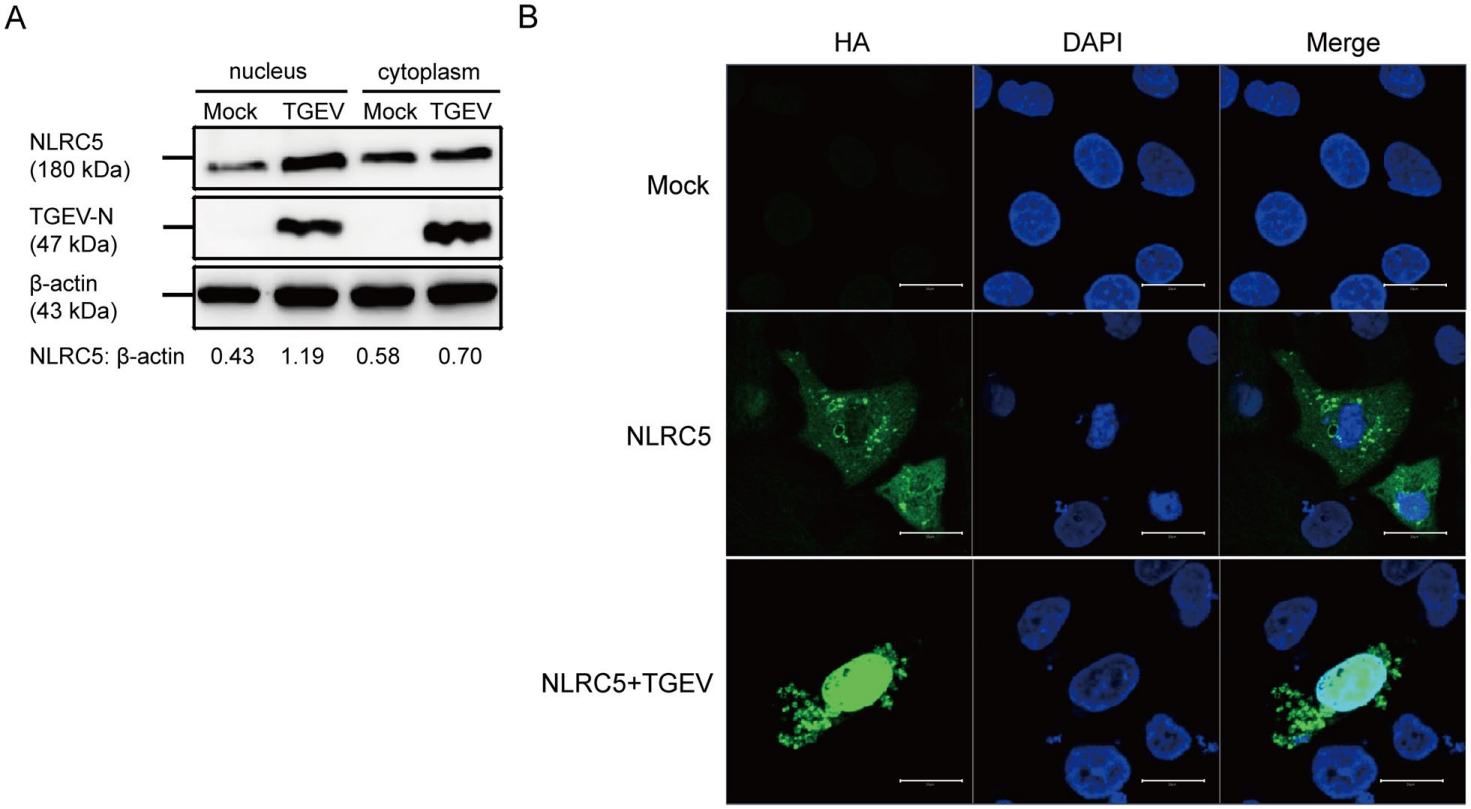
**TGEV promotes the nuclear transportation of NLRC5**. **A** ST cells were transfected with pCAGGS-HA-NLRC5 for 24 h, infected with TGEV (MOI = 1) and harvested at 24 hpi. The indicated proteins from the cell lysates of the nuclear and cytosolic fraction were analyzed by western blot. **B** The effect of TGEV on the cellular localization of HA-tagged NLRC5 in ST cells was examined through immunofluorescence analysis. Scale bar: 20 µm.

Nuclear localization of NLRC5 is indispensable for its role as a trans-activator of MHC-I. To investigate whether TGEV enhances NLRC5 activity by promoting its nuclear translocation, ST cells transfected with the recombinant plasmid pCAGGS-HA-NLRC5 were infected with TGEV for 24 h. Western blot analysis of nuclear fractions revealed a significant increase in NLRC5 protein levels following TGEV infection (Figure 4A). In addition, confocal microscopy was employed to examine NLRC5 subcellular localization. As shown in Figure 4B, the nuclear green fluorescence signal was significantly enhanced in TGEV-infected cells compared with uninfected controls. The results provide evidence that TGEV infection facilitates the nuclear translocation of NLRC5, thereby likely enhancing its transcriptional activity. These findings revealed a direct interaction between TGEV and NLRC5, suggesting that the viral modulation of NLRC5 localization as a potential mechanism to alter host antiviral responses.

### TGEV upregulates SLA-I and NLRC5 expression via RIG-I

**Figure 5:**
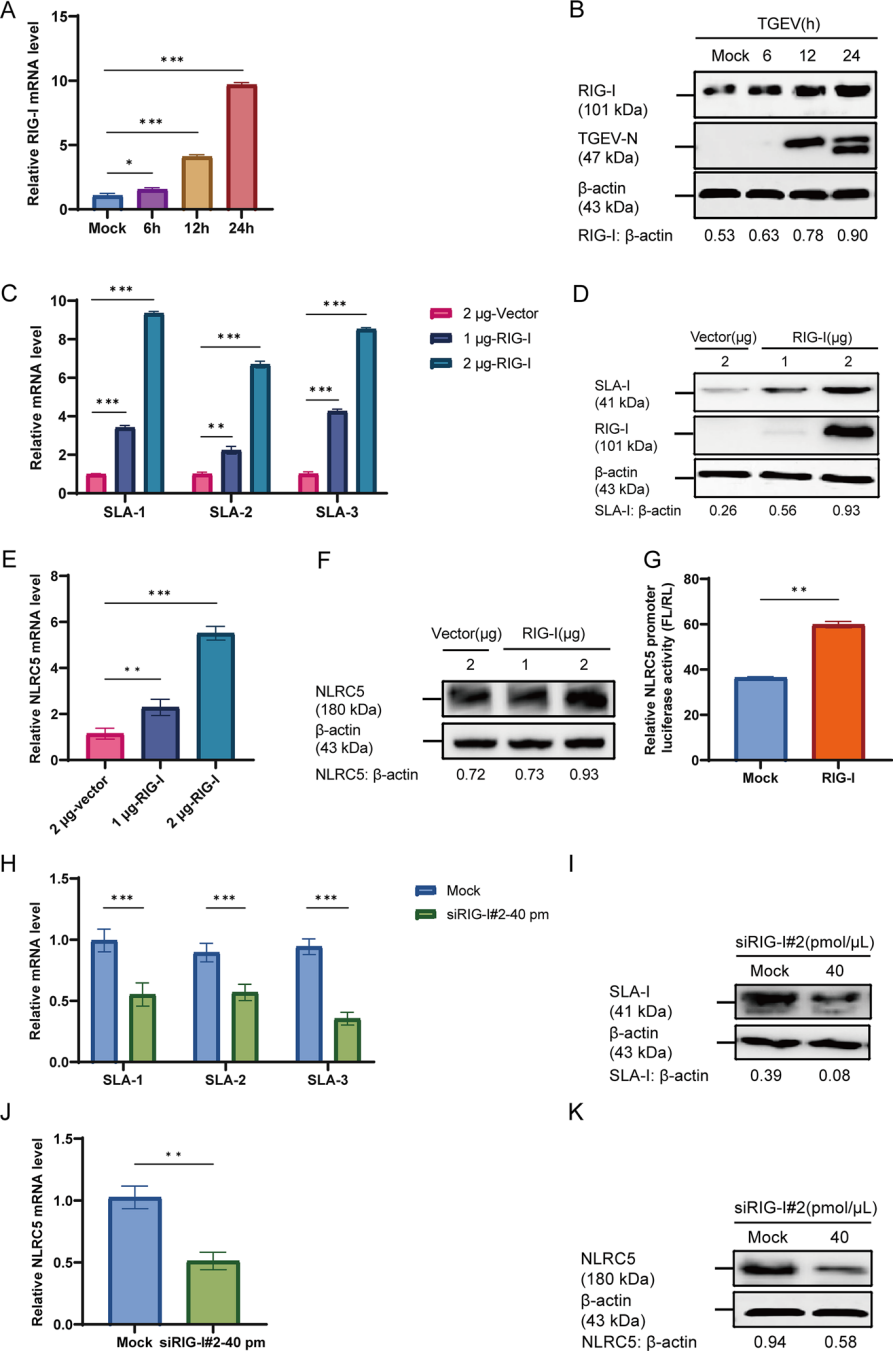
**TGEV upregulates RIG-I to increase the expression of SLA-I and NLRC5 (***n*** = 3)**. **A**, **B** ST cells were infected with TGEV (MOI = 1) and harvested at 6, 12, and 24 hpi. The mRNA levels (**A**) and protein levels (**B**) of RIG-I were assessed by RT-qPCR and western blot, respectively. **C**–**F** ST cells were transfected with pCAGGS-HA (2 μg) or pCAGGS-HA-RIG-I (1 or 2 μg). After 24 h, the cells were harvested for analysis. The relative mRNA levels of SLA-1, SLA-2, and SLA-3 (**C**) and NLRC5 (**E**) were quantified by RT-qPCR, while the protein levels of SLA-I (**D**) and NLRC5 (**F**) were analyzed by western blot. **G** ST cells were co-transfected with the NLRC5 promoter-driven dual-luciferase reporter plasmid (500 ng) and the pRL-TK plasmid (50 ng) for 24 h. Subsequently, the cells were transfected with pCAGGS-HA-RIG-I. Luciferase activity was then measured 24 h later using a dual-luciferase assay (*FL* firefly luciferase, *RL* Renilla luciferase). **H**–**K** ST cells were transfected with siRIG-I#2 at 40 pmol/μL for 24 h and then infected with TGEV. After an additional 24 h, the relative mRNA levels of SLA-1, SLA-2, and SLA-3 (**H**) and NLRC5 (**J**) were analyzed by RT-qPCR, while the protein levels of SLA-I (**I**) and NLRC5 (**K**) were assessed by western blot. *p < 0.05, **p < 0.01, ***p < 0.001.

RIG-I functions as an essential sensor in innate immunity that recognizes viral RNA and triggers IFN-I production and inflammatory signaling, forming the primary host defense mechanism [20]. Initially, we assessed RIG-I expression in ST cells at 6, 12, and 24 hpi with TGEV. Both mRNA and protein levels of RIG-I peaked at 24 hpi (Figures 5A, B), revealing a time-dependent upregulation of RIG-I expression during TGEV infection. Subsequently, we constructed the recombinant plasmid pCAGGS-HA-RIG-I, which was successfully expressed in ST cells, as confirmed by western blot (Additional file 2A). To investigate whether RIG-I modulates SLA-I and NLRC5 expression, ST cells were transfected with 2 μg of the pCAGGS-HA or with increasing doses of the recombinant plasmid pCAGGS-HA-RIG-I (1 μg or 2 μg), followed by culture for 24 h. RT-qPCR analysis showed that transfection with 2 μg of recombinant plasmid pCAGGS-HA-RIG-I significantly elevated mRNA levels of SLA-1, SLA-2, SLA-3, and NLRC5 compared with the vector group (*p* < 0.001) (Figures 5C, E). Consistent with the results of RT-qPCR, western blot confirmed dose-dependent upregulation of SLA-I and NLRC5 proteins in response to RIG-I overexpression (Figure 5D, F). To explore whether RIG-I affects the transcription of NLRC5, ST cells were co-transfected with recombinant plasmid pCAGGS-HA-RIG-I and NLRC5 promoter driven luciferase reporter plasmid. After 24 h, luciferase activity was measured and showed a significant increase in the RIG-I-transfected group compared with the mock-transfected control (*p* < 0.01) (Figure 5G), indicating that RIG-I enhanced NLRC5 promoter activity. To investigate whether RIG-I upregulates SLA-I expression via NLRC5 activation, we identified siRIG-I#2 (40 pmol/μL) as the most effective siRNA, which significantly suppressed RIG-I expression relative to the mock group (*p* < 0.001) (Additional file 2B and C). ST cells were transfected with siRIG-I#2 (40 pmol/μL) for 24 h, followed by TGEV infection for an additional 24 h. Analysis by RT-qPCR and western blot revealed that RIG-I silencing significantly downregulated both mRNA and protein levels of SLA-I and NLRC5 compared with the control group (Figures 5H–K), indicating that RIG-I silencing suppresses the expression of SLA-I and NLRC5.

### IFN-β enhances the upregulation of SLA-I and NLRC5

**Figure 6:**
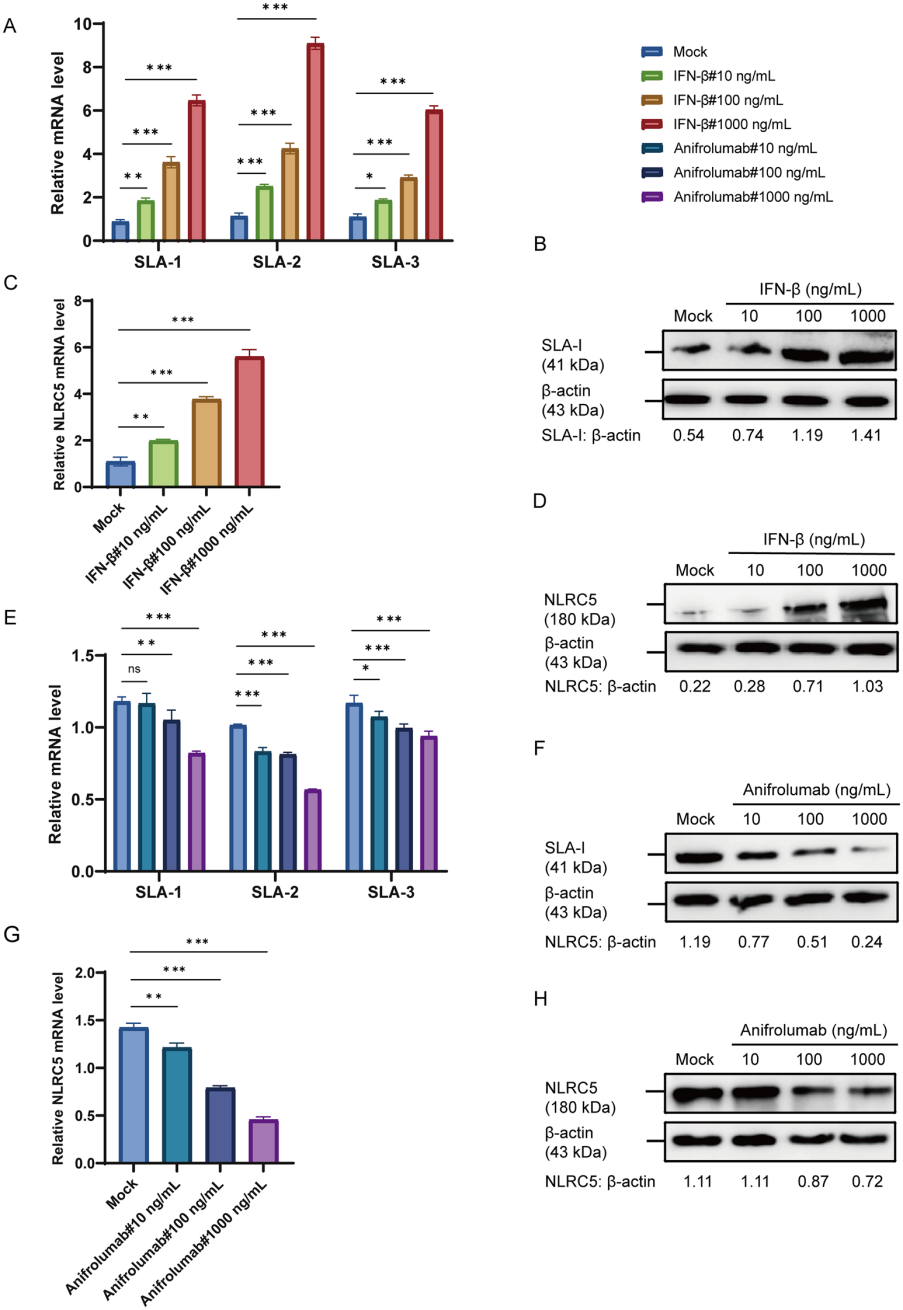
**IFN-β promotes the expression of SLA-I and NLRC5**. **A**–**D** ST cells were treated with 10, 100, and 1000 ng/mL of IFN-β, and cell samples were harvested 24 h later. The relative mRNA levels of SLA-1, SLA-2, SLA-3 (**A**), and NLRC5 (**C**) were accessed by RT-qPCR. Subsequently, the protein levels of SLA-I (**B**) and NLRC5 (**D**) were analyzed by western blot. **E**–**H** ST cells were treated with 10, 100, and 1000 ng/mL of Anifrolumab, and cell samples were harvested 24 h later. The relative mRNA levels of SLA-1, SLA-2, SLA-3 (**E**), and NLRC5 (**G**) were accessed by RT-qPCR. Subsequently, the protein levels of SLA-I (**F**) and NLRC5 (**H**) were analyzed by western blot. *p < 0.05, **p < 0.01, ***p < 0.001.

To assess the impact of IFN-I signaling in SLA-I and NLRC5 expression, ST cells were treated with ten-fold serial dilution of IFN-β (10, 100, and 1000 ng/mL) for 24 h, and SLA-I and NLRC5 were analyzed for their expression levels. The results showed that both the transcriptional levels (Figures 6A, C) and translational levels (Figures 6B, D) of SLA-I and NLRC5 progressively increased with higher concentrations of IFN-β. To further validate the results, ST cells were treated for 24 h with serial tenfold dilutions of Anifrolumab (10, 100, and 1000 ng/mL) to inhibit the activity of IFN-I, and the expression levels of SLA-I and NLRC5 were analyzed. The results showed that both the transcriptional levels (Figures 6E, G) and translational levels (Figures 6F, H) of SLA-I and NLRC5 progressively reduced with higher concentrations of Anifrolumab. These results showed that IFN-β enhances the upregulation of SLA-I and NLRC5 expression in ST cells.

### TGEV enhances SLA-I and NLRC5 expression via IFN-β/STAT1

**Figure 7:**
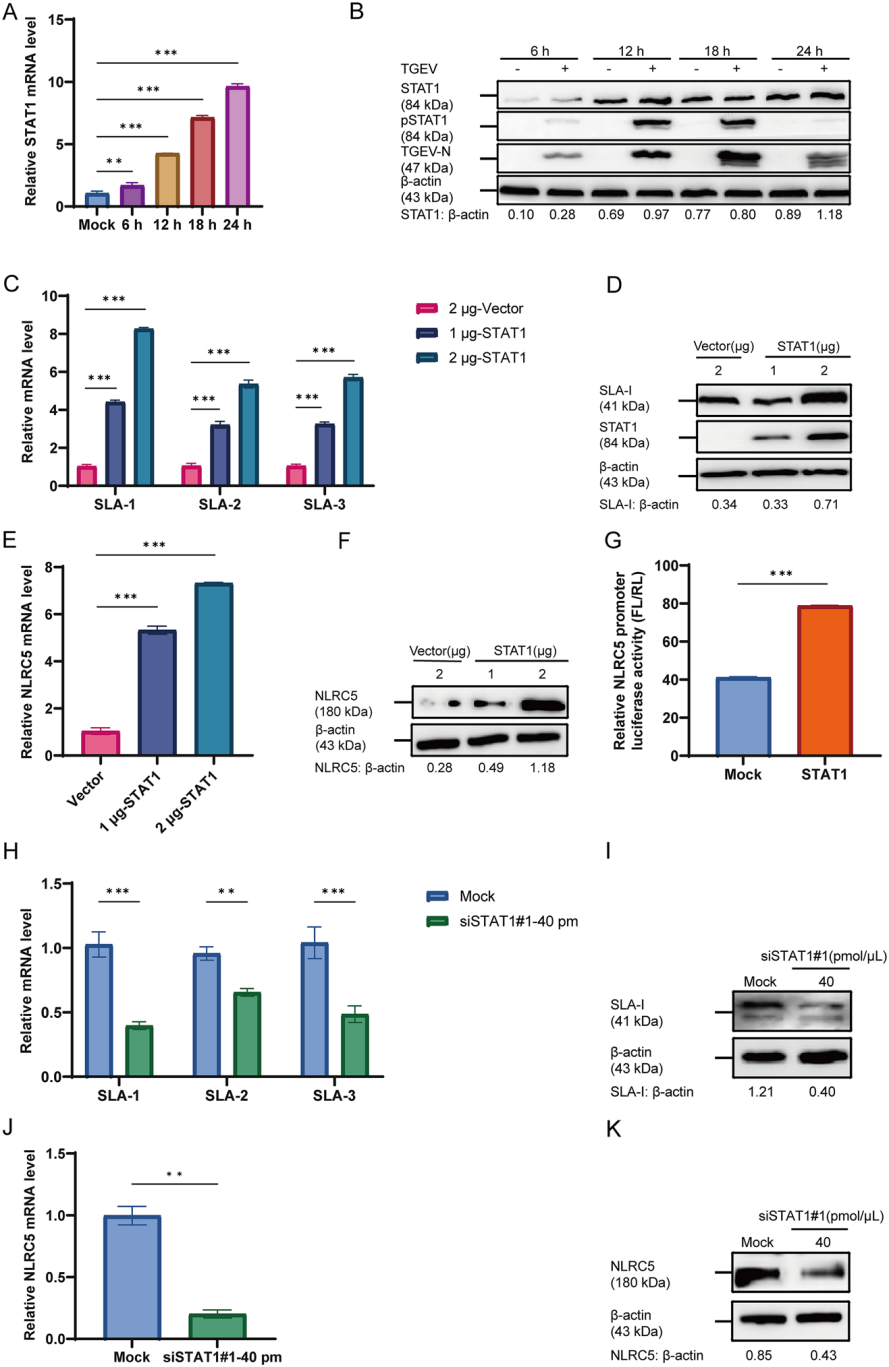
**TGEV upregulates SLA-I and NLRC5 production through activation of IFN-β-mediated STAT1 signaling (***n*** = 3)**. **A**, **B** ST cells were infected with TGEV (MOI = 1) and harvested at 6, 12, 18, and 24 hpi. The mRNA level (**A**) and protein phosphorylation level (**B**) of STAT1 were assessed by RT-qPCR and western blot, respectively. **C**–**F** ST cells were transfected with pCAGGS-FLAG (2 μg) or pCAGGS-FLAG-STAT1 (1 or 2 μg). After 24 h, the cells were harvested for analysis. The relative mRNA levels of SLA-1, SLA-2, and SLA-3 (**C**) and NLRC5 (**E**) were quantified by RT-qPCR, while the protein levels of SLA-I (**D**) and NLRC5 (**F**) were analyzed by western blot. **G** ST cells were co-transfected with the NLRC5 promoter-driven dual-luciferase reporter plasmid (500 ng) and the pRL-TK plasmid (50 ng). After 24 h, cells were transfected with pCAGGS-FLAG-STAT1, and luciferase activity was measured 24 h later using a dual-luciferase assay (FL: firefly luciferase, RL: Renilla luciferase). **H**–**K** ST cells were transfected with siSTAT1#1 at 40 pmol/μL for 24 h and then infected with TGEV (MOI = 1). After an additional 24 h, the relative mRNA levels of SLA-1, SLA-2, and SLA-3 (**H**) and NLRC5 (**J**) were analyzed by RT-qPCR, while the protein levels of SLA-I (**I**) and NLRC5 (**K**) were detected by western blot. *p < 0.05, **p < 0.01, ***p < 0.001.

To investigate the role of STAT1 in mediating TGEV-induced upregulation of SLA-I, ST cells were infected with TGEV and samples were collected at 6, 12, 18, and 24 hpi. The results demonstrated that the mRNA levels of STAT1 peaked at 24 hpi (*p* < 0.001) (Figure 7A). Concurrently, the phosphorylation level of STAT1 was elevated as early as 6 hpi and declined by 24 hpi (Figure 7B). Subsequently, the recombinant plasmid pCAGGS-FLAG-STAT1 was constructed and successfully expressed in ST cells (Additional file 3A). To assess the impact of STAT1 on SLA-I and NLRC5 expression, ST cells were transfected with 1 or 2 μg of the pCAGGS-FLAG-STAT1, and samples were collected after 24 h. The recombinant plasmid of pCAGGS-FLAG-STAT1 with 2 μg significantly elevated both mRNA (Figures 7C, E) and protein (Figures 7D, F) levels of SLA-I and NLRC5 compared with the vector group (*p* < 0.001). Furthermore, luciferase activity of NLRC5 promoter was measured at 24 h post-transfection. Compared with the mock group, a marked elevation in luciferase activity was observed, indicating that STAT1 activated the NLRC5 promoter (*p* < 0.001) (Figure 7G). To explore whether STAT1 upregulates SLA-I expression by activating NLRC5, siSTAT1#1 with 40 pmol/μL was screened out to inhibit STAT1 expression (Additional file 3B and C). Furthermore, the functions of STAT1 on the expression of SLA-I and NLRC5 was measured. ST cells were transfected with siSTAT1#1 (40 pmol/μL) for 24 h, followed by TGEV infection for an additional 24 h, then cells were harvested for RT-qPCR and western blot analysis of SLA-I and NLRC5 expression. These results showed that STAT1 deficiency leads to significant downregulation of both SLA-I and NLRC5 at mRNA and protein levels (Figures 7H–K).

### TGEV ORF7 protein enhances the expression of SLA-I and NLRC5

**Figure 8:**
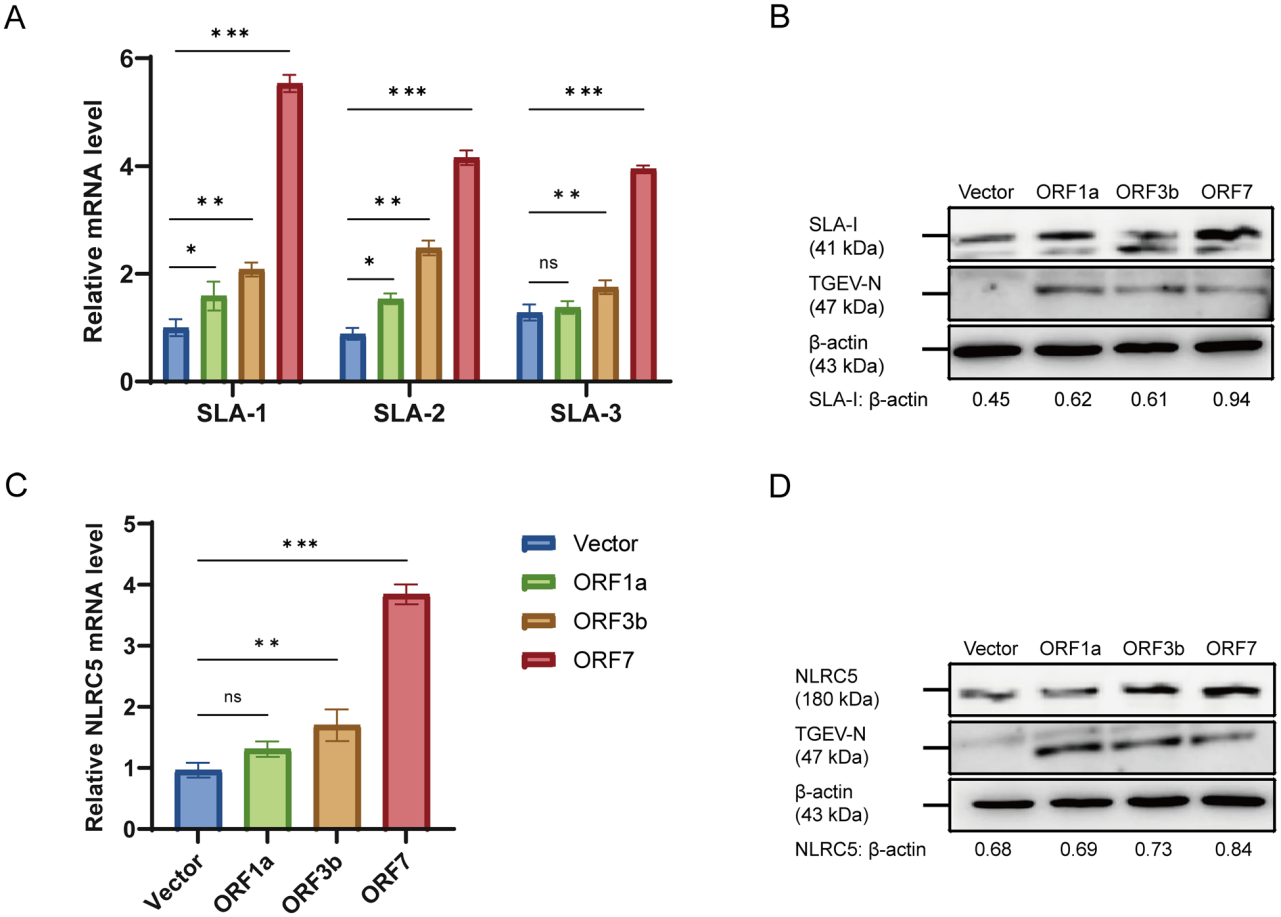
**ORF7 upregulates the expression of SLA-I and NLRC5 (***n*** = 3)**. **A**–**D** ST cells were transfected with pCAGGS-HA-ORF1a, pCAGGS-HA-ORF3b, and pCAGGS-HA-ORF7. At 24 h post-transfection, the cells were harvested. The relative mRNA levels of SLA-1, SLA-2, and SLA-3 (**A**) and NLRC5 (**C**) were quantified by RT-qPCR. Subsequently, the protein levels of SLA-I (**B**) and NLRC5 (**D**) were analyzed by western blot. *p < 0.05, **p < 0.01, ***p < 0.001.

Since TGEV replication is essential for upregulating both SLA-I and NLRC5, so we confirmed the role of specific TGEV nonstructural proteins. Expression of recombinant plasmids encoding TGEV ORF1a, ORF3b, and ORF7 was confirmed in ST cells (Additional file 4). Each plasmid was then individually transfected into ST cells, and cells were harvested 24 h later for analysis of SLA-I and NLRC5 expression. RT-qPCR analysis showed that ORF7 significantly promoted the mRNA levels of SLA-1, SLA-2, and SLA-3 (*p* < 0.001) (Figure 8A), and the western blot analysis was performed to further confirmed the protein level of SLA-I (Figure 8B). Similarly, ORF7 upregulated NLRC5 expression at both mRNA (*p* < 0.001) and protein levels (Figures 8C, D), suggesting that the TGEV ORF7 protein serves as a core regulator in the induction of SLA-I and NLRC5.

## Discussion

Porcine coronaviruses have caused significant economic losses in the global swine industry in recent years, with the coinfection involving these viruses frequently observed in clinical cases [34–36]. Among them, TGEV predominantly infects suckling piglets within the first 2 weeks of life, causing extensive necrosis of jejunal and ileal epithelial cells. This damage leads to a marked decline in the activity of key intestinal enzymes such as alkaline phosphatase and lactase [37]. TGEV infects host intestinal epithelial cells by binding its S protein to porcine aminopeptidase N (pAPN) on the cell surface [38]. Sialic acids further enhance viral adhesion and facilitate penetration through the intestinal mucus barrier [39]. The TGEV genome encodes several nonstructural proteins that cooperate with host factors, including TMEM41B, to form double membrane vesicles essential for viral replication [5, 40]. TGEV induces apoptosis through both the death receptor-mediated extrinsic pathway and the mitochondrial-mediated intrinsic pathway, leading to intestinal epithelial cell death and villous atrophy [41]. In addition, the viral Nsp1 disrupts host protein synthesis, while other proteins such as Nsp2 activate the NF-κB pathway, thereby triggering a proinflammatory response [42, 43]. Variations in TGEV have been associated with mutations in the S protein and the ORF3 region. Some recombinant strains exhibit altered tissue tropism due to these genetic changes [44]. The ORF7 protein, encoded at the 3′ terminus of the viral genome, serves as a genetic marker for phylogenetic analyses [9]. Emerging evidence further indicates that TGEV possesses potential for cross-species transmission, underscoring the critical need for effective preventive measures against this pathogen [45–47].

MHC-I, which is stably located on the surface of all nucleated cells, is critically involved in immune defense by presenting intracellular antigens to CD8^+^ T lymphocytes, resulting in the elimination of infected or abnormal cells [28]. NLRC5 serves as a major transcriptional regulator of MHC-I expression, functioning as a trans-activator that specifically interacts with conserved proximal cis-regulatory elements within MHC-I promoters to initiate their transcription, which is vital for the activation of CD8^+^ T cells [28, 29]. Our initial investigations revealed that TGEV infection markedly upregulates SLA-I expression both in vivo and in vitro*,* with SLA-I levels increasing in a time-dependent manner during infection (Figure 1). Correspondingly, NLRC5 expression also showed progressive upregulation in TGEV-infected ST cells. Moreover, overexpression of NLRC5 led to a dose-dependent enhancement of SLA-I protein levels. Crucially, we definitively identified that TGEV-induced SLA-I upregulation requires the activation of NLRC5 (Figure 2), which is consistent with the report that porcine deltacoronavirus (PDCoV) upregulates SLA-I expression via NLRC5-dependent mechanisms [22]. However, PEDV exhibited a unique regulatory pattern where it enhanced NLRC5 transcription while simultaneously suppressing its translation, ultimately preventing SLA-I upregulation [18]. Furthermore, SARS-CoV-2 infection leads to a suppression of MHC-I expression [15]. In addition, we identified that NLRC5 exerts antiviral activity by suppressing TGEV replication (Figure 3), while TGEV promotes NLRC5 nuclear translocation (Figure 4). These findings indicate that although these viruses all belong to the *coronavirus* family, their strategies for modulating MHC-I expression differ substantially, reflecting distinct virus–host interaction mechanisms.

RIG-I serves as a crucial PRR capable of detecting diverse viral pathogens. Through its recognition of viral RNA, RIG-I activates downstream signaling cascades that induce IFN-I and proinflammatory cytokine production, thereby orchestrating robust antiviral host defenses [48]. In the present study, the observed upregulation of IFN-stimulated genes aligns with this canonical antiviral signaling paradigm (Figure 6). The established pathway involves the cytoplasmic sensor RIG-I signaling through the mitochondrial antiviral-signaling protein (MAVS) to coordinately activate the key transcription factors IRF3 and NF-κB, which are indispensable for IFN-β gene transcription [49]. In parallel to the RIG-I-like receptor (RLR) pathway, Toll-like receptors (TLRs) signaling can also robustly induce NF-κB and promote IRF3 phosphorylation, thereby amplifying the IFN-I response. This regulatory mechanism remains operational under specific conditions, including NLRC5 deficiency, a finding of direct relevance to our investigation [26]. This signaling framework is conserved across coronavirus infections. For instance, RLR-mediated NF-κB activation has been documented during TGEV infection [50], and PDCoV have been shown to induce IFN-β production via the RIG-I and IRF1 pathway [22]. Collectively, these established mechanisms provide a plausible framework for the signaling events initiated by TGEV in our model, potentially mediated through specific viral proteins such as the accessory protein ORF7. IFN constitute a critical family of cytokines that suppress viral replication by interfering with viral transcription and translation [51]. Following secretion, IFN-I engages specific cell surface receptors, initiating a signaling cascade that primarily activates the Janus Kinase (JAK)-STAT signaling pathway [52]. Notably, STAT1, a key transcription factor downstream of this pathway, possesses binding sites within the promoter region of NLRC5 [27], suggesting a direct regulatory link. In this study, we observed a time-dependent increases in the RIG-I expression in TGEV-infected ST cells in Figure 5, which correspond to a dose-dependent upregulation of both SLA-I and NLRC5 that correlated with RIG-I expression levels, and this upregulation is mediated by RIG-I. Moreover, we observed that both the transcription and translation levels of SLA-I and NLRC5 were progressively enhanced by increasing concentrations of IFN-β. This finding is consistent with previous research on PDCoV showing that RIG-I upregulates MHC-I [22]. In addition, as shown in Figure 7, our results indicated that TGEV infection induced STAT1 phosphorylation, and the STAT1 expression increased in a time-dependent manner as TGEV infection progressed. Crucially, both SLA-I and NLRC5 expression exhibited STAT1 dose-dependent upregulation, mechanistically linked to STAT1-mediated transcriptional activation. However, the interplay between coronaviruses and host STAT signaling exhibits notable complexity and divergence. For instance, SARS-CoV-2 targets the STAT1–IRF1–NLRC5 axis to suppress MHC-I [53], and the tyrosine phosphatase nonreceptor type 14 inhibits STAT3 activation in PEDV [54], which imply the complexity of the interaction between coronaviruses and their hosts.

In our study, upregulation of SLA-I was specifically induced by live TGEV but not by inactivated virus, suggesting a critical role for viral nonstructural proteins. To further clarify the underlying mechanism, we systematically evaluated the roles of selected TGEV nonstructural proteins in regulating SLA-I and NLRC5 expression. Since the HN-2012 strain is a ORF3a deficiency variant, ORF3b protein was selected for investigation. We focused our screening on ORF1a, ORF3b, and ORF7 of TGEV (Figure 8) based on both functional relevance and strain‑specific genetic characteristics. In other porcine enteric coronaviruses, PEDV Nsp1 protein suppresses NLRC5 translation to evade MHC-I-mediated immunity [18], while the Nsp1 of coronaviruses that encoded within ORF1a/b functions as a key virulence determinant [31]. Although ORF1a may participate in immune modulation, we focused on ORF1b for this initial analysis owing to its indispensable and highly conserved function as the core component of the viral replication machinery [55, 56]. Meanwhile, SARS-CoV-2 utilizes distinct strategies to evade cellular immunity. The viral accessory proteins ORF3a and ORF7a suppress MHC-I expression through independent mechanisms, and the MHC-I-like domain within the S protein directly binds T cell receptors, thereby competitively inhibiting recognition of endogenous MHC-I-peptide complexes [15, 57]. The data identify TGEV ORF7 as a key viral factor responsible for the upregulation of both SLA-I and NLRC5, providing new insight into how TGEV modulates host antigen presentation during infection.

**Figure 9:**
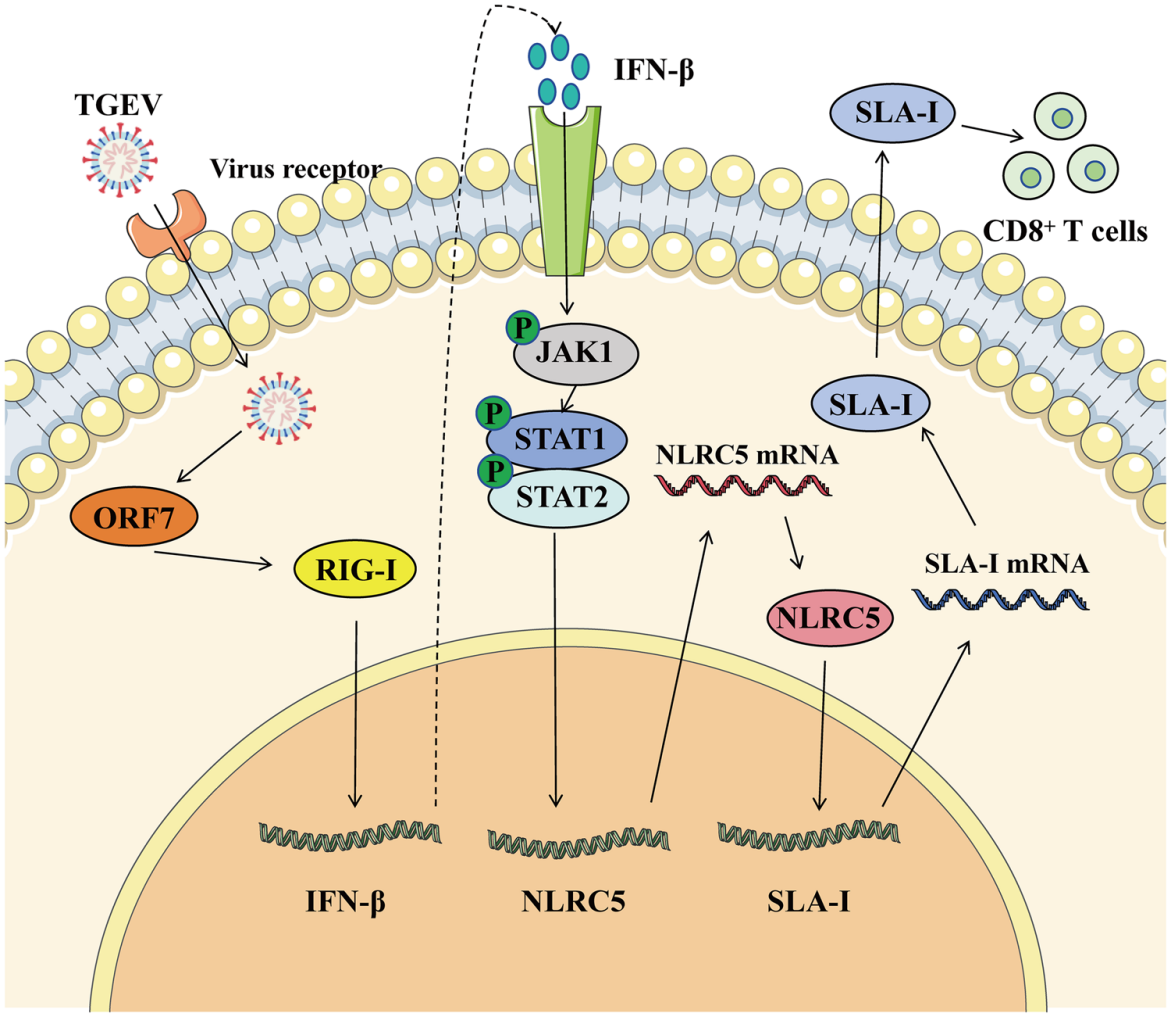
**Schematic illustration of TGEV activates RIG-I/IFN-β/STAT1 axis to promote NLRC5-mediated SLA-I upregulation**. TGEV infection significantly upregulates SLA-I expression, a process mediated by the virus-induced upregulation of NLRC5. TGEV activates the cytoplasmic RNA sensor RIG-I, triggering a signaling cascade that leads to IFN-β production and subsequent STAT1 phosphorylation. The activated STAT1 then transcriptionally upregulates NLRC5, which in turn drives the expression of SLA-I and its antigen-processing components. The resulting enhancement of SLA-I surface expression contributes to the host antiviral response by inhibiting viral proliferation. Notably, the TGEV ORF7 protein plays a pivotal role in upregulating both NLRC5 and SLA-I during infection. This work delineates the RIG-I/IFN-β/STAT1 signaling pathway as a critical host–TGEV interaction mediator. *Green circles* marked with “p” designate the phosphorylated state of the corresponding proteins.

In conclusion, TGEV infection significantly upregulates the expression of SLA-I, a process mediated by TGEV-induced NLRC5 upregulation. We identified the RIG-I/IFN-β/STAT1 signaling pathway as a critical host-TGEV interaction mediator, with the TGEV ORF7 protein playing a pivotal role in NLRC5 and SLA-I upregulation during infection (Figure 9). Mechanistically, TGEV activates the RIG-I/IFN-β/STAT1 axis to drive NLRC5-mediated SLA-I expression, which in turn inhibits viral proliferation. These findings elucidate a novel immune regulatory mechanism that balances viral replication and host antiviral defense, offering insights for targeted control strategies against porcine enteric coronaviruses.

## Supplementary Information

**Additional file 1:** **Overexpression and knockdown of NLRC5 in ST cells.** ST cells were transfected with the pCAGGS-HA-NLRC5 and collected at 24 h post-transfection. The expression of pCAGGS-HA-NLRC5 was confirmed by western blot. ST cells were treated with siNLRC5 at concentrations of 10, 20, 30, and 40 pmol/μL. After 24 h, the expression of NLRC5 was detected by RT-qPCR and western blot. *p < 0.05, **p < 0.01, ***p < 0.001.

**Additional file 2: Overexpression and knockdown of RIG-I in ST cells. **ST cells were transfected with the pCAGGS-HA-RIG-I and collected at 24 h post-transfection. The expression of pCAGGS-HA-RIG-I was confirmed by western blot. ST cells were treated with siRIG-I at concentrations of 20, 40, and 60 pmol/μL. After 24 h, the expression of NLRC5 was detected by RT-qPCR and western blot. *p < 0.05, **p < 0.01, ***p < 0.001.

**Additional file 3: Overexpression and knockdown of STAT1 in ST cells.** ST cells were transfected with the pCAGGS-FLAG-STAT1 and harvested at 24 h post-transfection. The expression of pCAGGS-FLAG-STAT1 was confirmed by western blot. ST cells were treated with siSTAT1 at concentrations of 10, 20, 40, and 60 pmol/μL. After 24 h, the expression of STAT1 was detected by RT-qPCR and western blot. *p < 0.05, **p < 0.01, ***p < 0.001.

**Additional file 4: Expression of ORF proteins of TGEV in ST cells.** The expression of pCAGGS-HA-ORF1a was detected by western blot. The expression of pCAGGS-HA-ORF3b was detected by western blot. The expression of pCAGGS-HA-ORF7 was detected by western blot.

## Data Availability

The raw data supporting the conclusions of this article will be made available from the corresponding author upon reasonable request.
