# Supplementary figures and images for: TGEV activates RIG-I/IFN-β/STAT1 axis to promote NLRC5-mediated SLA-I upregulation
Source: Vet Res. 2026 Mar 9;57:51. doi: 10.1186/s13567-026-01715-z (PMC13085429; doi:10.1186/s13567-026-01715-z)

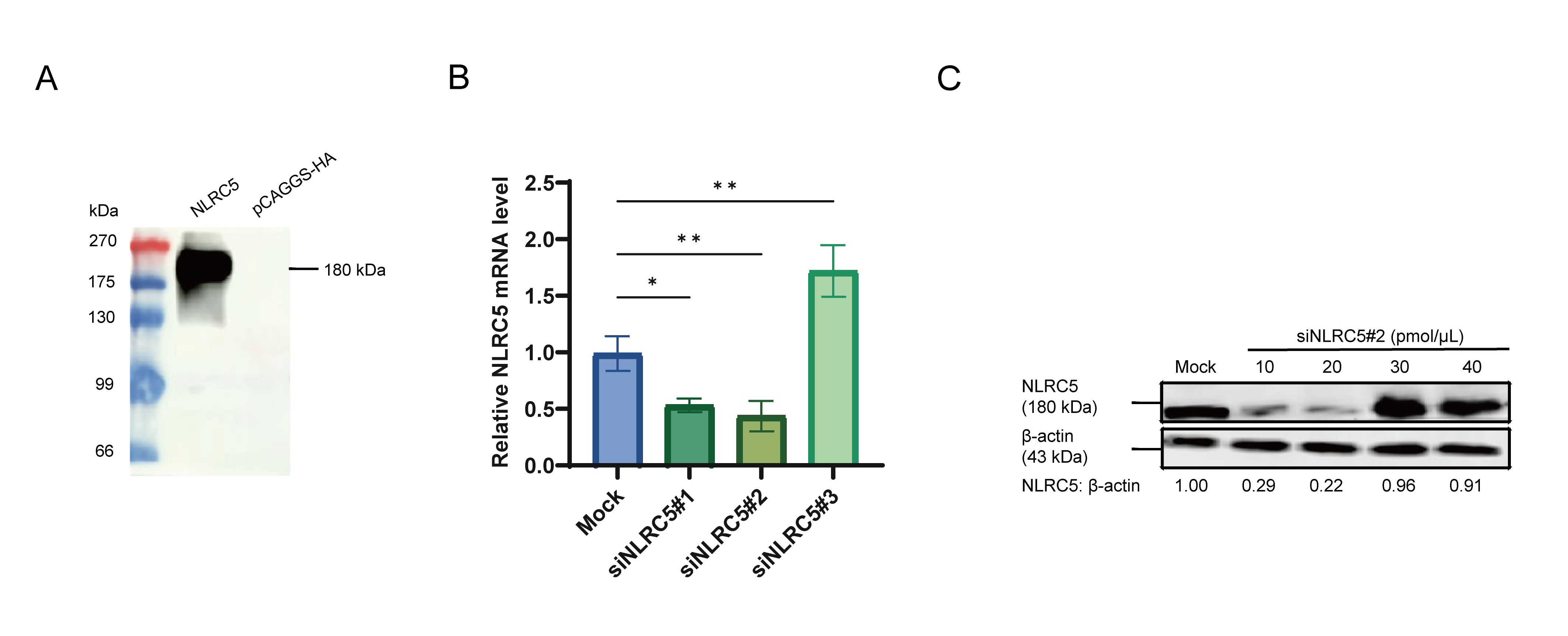

Supplement: Supplementary file 1 — Additional file 1: Overexpression and knockdown of NLRC5 in ST cells. ST cells were transfected with the pCAGGS-HA-NLRC5 and collected at 24 h post-transfection. The expression of pCAGGS-HA-NLRC5 was confirmed by western blot. ST cells were treated with siNLRC5 at concentrations of 10, 20, 30, and 40 pmol/μL. After 24 h, the expression of NLRC5 was detected by RT-qPCR and western blot. *p < 0.05, **p < 0.01, ***p < 0.001. [file 13567_2026_1715_MOESM1_ESM.tif]

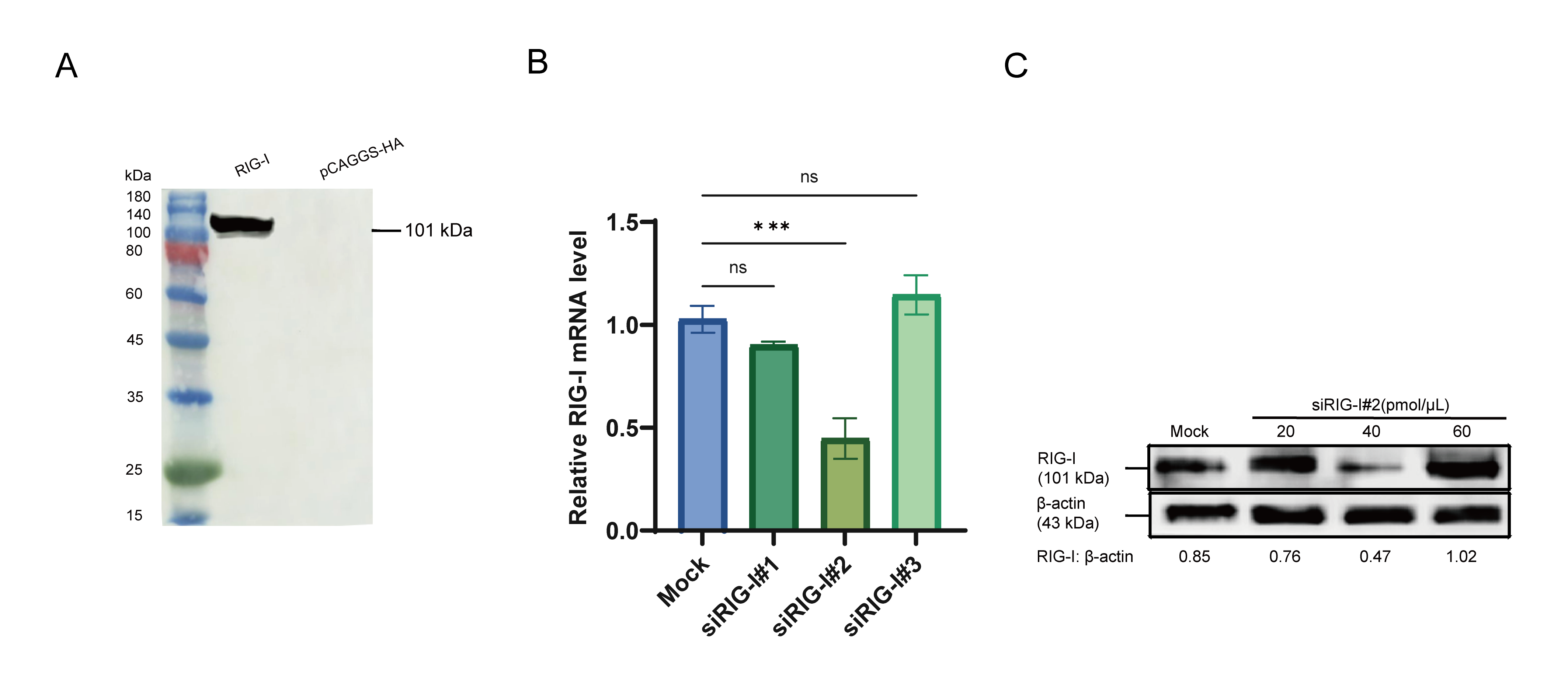

Supplement: Supplementary file 2 — Additional file 2: Overexpression and knockdown of RIG-I in ST cells. ST cells were transfected with the pCAGGS-HA-RIG-I and collected at 24 h post-transfection. The expression of pCAGGS-HA-RIG-I was confirmed by western blot. ST cells were treated with siRIG-I at concentrations of 20, 40, and 60 pmol/μL. After 24 h, the expression of NLRC5 was detected by RT-qPCR and western blot. *p < 0.05, **p < 0.01, ***p < 0.001. [file 13567_2026_1715_MOESM2_ESM.tif]

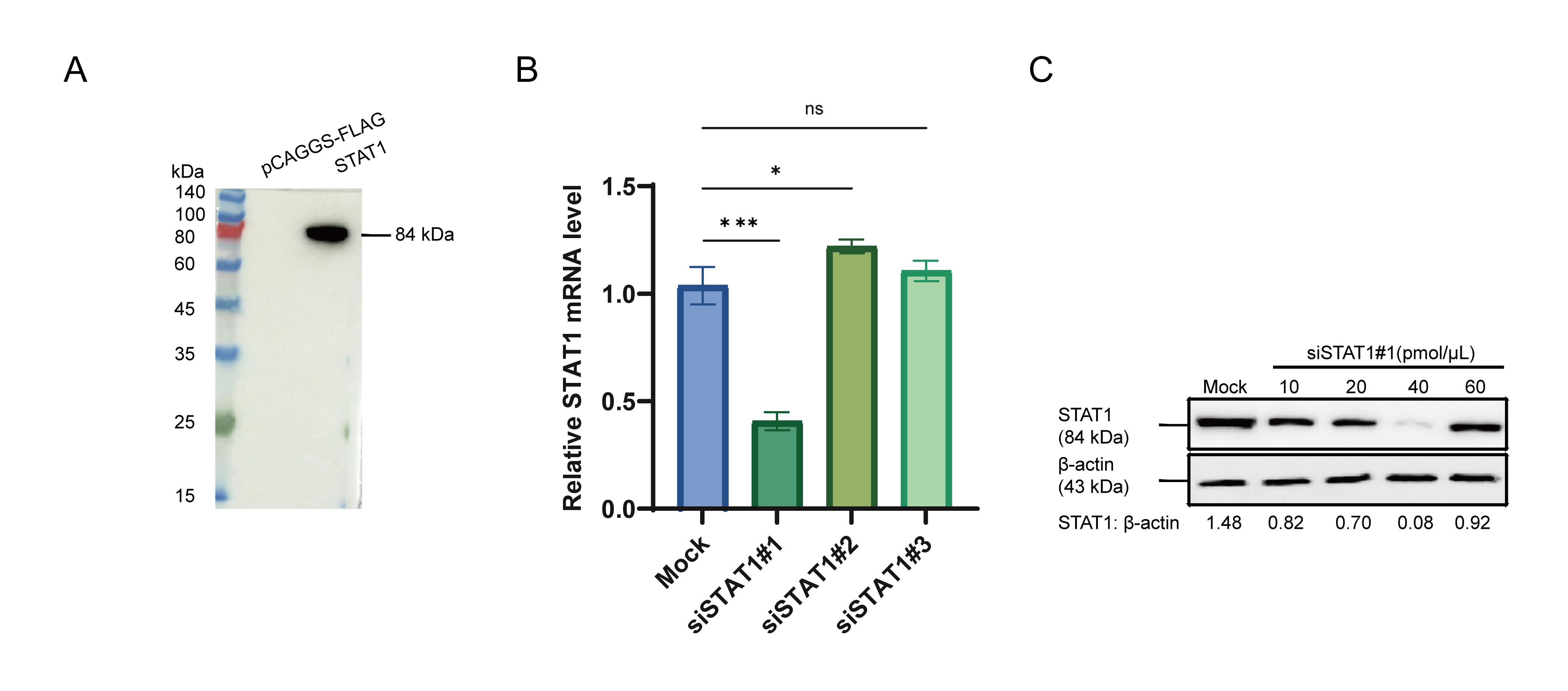

Supplement: Supplementary file 3 — Additional file 3: Overexpression and knockdown of STAT1 in ST cells. ST cells were transfected with the pCAGGS-FLAG-STAT1 and harvested at 24 h post-transfection. The expression of pCAGGS-FLAG-STAT1 was confirmed by western blot. ST cells were treated with siSTAT1 at concentrations of 10, 20, 40, and 60 pmol/μL. After 24 h, the expression of STAT1 was detected by RT-qPCR and western blot. *p < 0.05, **p < 0.01, ***p < 0.001. [file 13567_2026_1715_MOESM3_ESM.tif]

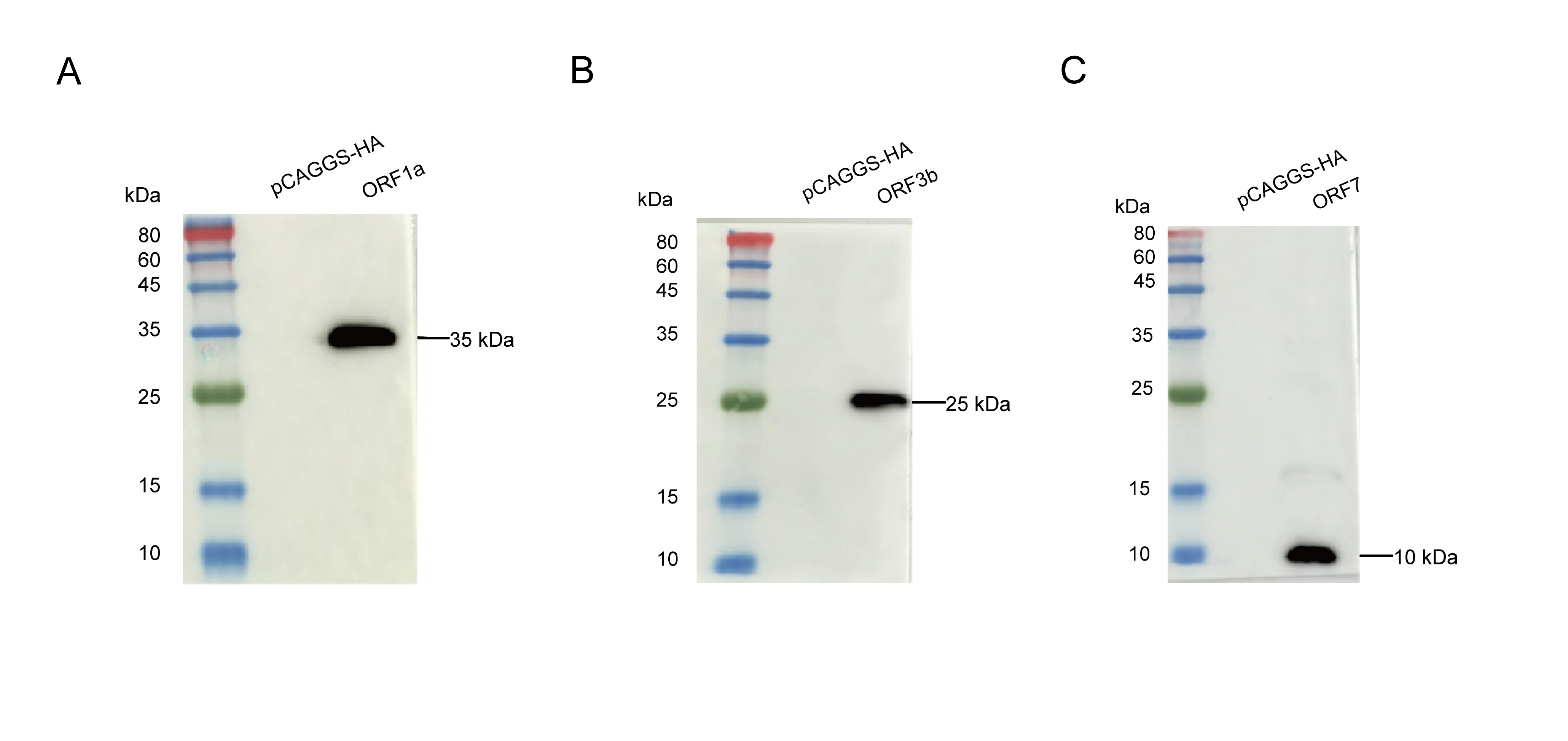

Supplement: Supplementary file 4 — Additional file 4: Expression of ORF proteins of TGEV in ST cells. The expression of pCAGGS-HA-ORF1a was detected by western blot. The expression of pCAGGS-HA-ORF3b was detected by western blot. The expression of pCAGGS-HA-ORF7 was detected by western blot. [file 13567_2026_1715_MOESM4_ESM.tif]
